# Lepidoptera of Canada

**DOI:** 10.3897/zookeys.819.27259

**Published:** 2019-01-24

**Authors:** Gregory R. Pohl, Jean-François andry, B. Chris Schmidt, Jeremy R. deWaard

**Affiliations:** 1 Natural Resources Canada, Canadian Forest Service, 5320 – 122 St., Edmonton, Alberta, T6H 3S5, Canada Natural Resources Canada Edmonton Canada; 2 Agriculture and Agri-Food Canada, Ottawa Research and Development Centre, 960 Carling Avenue, Ottawa, Ontario, K1A 0C6, Canada Agriculture and Agri-Food Canada Ottawa Canada; 3 Centre for Biodiversity Genomics, University of Guelph, 50 Stone Road East, Guelph, Ontario, N1G 2W1, Canada University of Guelph Guelph Canada

**Keywords:** biodiversity assessment, Biota of Canada, moths, butterflies

## Abstract

The known Lepidoptera (moths and butterflies) of the provinces and territories of Canada are summarised, and current knowledge is compared to the state of knowledge in 1979. A total of 5405 species are known to occur in Canada in 81 families, and a further 50 species have been reported but are unconfirmed. This represents an increase of 1348 species since 1979. The DNA barcodes available for Canadian Lepidoptera are also tabulated, based on a dataset of 148,314 specimens corresponding to 5842 distinct clusters. A further yet-undiscovered 1400 species of Lepidoptera are estimated to occur in Canada. The Gelechioidea are the most poorly known major lineage of Lepidoptera in Canada. Nunavut, Prince Edward Island, and British Columbia are thought to show the greatest deficit in our knowledge of Lepidoptera. The unglaciated portions of the Yukon (Beringia), and the Pacific Maritime, Montane Cordillera, and Western Interior Basin ecozones of British Columbia are also identified as hotbeds of undescribed biodiversity.

## Introduction

The order Lepidoptera (moths and butterflies) comprises the fourth-largest insect order in terms of global diversity, with approximately 158,000 described species (van Nieukerken et al. 2011), and an estimated total global diversity of 300,000 to 400,000 species (Kristensen et al. 2007). Butterflies are much better known than moths, but moth species outnumber butterfly species by at least 10 to one.

The higher classification of Lepidoptera is reasonably well known, thanks in large part to recent genetic work (Regier et al. 2012, Sohn et al. 2013, Heikkilä et al. 2014, Regier et al. 2015a, b, Sohn et al. 2016, Kawahara et al. 2017). An excellent summary of the higher classification of the order was presented by Aarvik et al. (2017). Presently 127 families of Lepidoptera are recognised globally (van Nieukerken et al. 2011). There is no comprehensive catalogue of the order, although catalogues of some constituent taxa exist, as well as regional works.

There has been considerable taxonomic work on Lepidoptera in Canada, but it has been scattered among hundreds of published works. Munroe (1979) provided an overview of major work up to that point, presented a rough count of 4692 known Canadian species, and included a prediction of 2042 undiscovered species, for a total fauna estimated to be 6734 species. Specific numbers were given for each family, which suggests that a precise count of species had been done. At that time, there was no comprehensive list of Lepidoptera of Canada, so Munroe probably relied on major taxonomic works and specimens in the Canadian National Collections of Insects, Arthropods and Nematodes (CNC).

Since Munroe (1979), we have learned a great deal about Canadian Lepidoptera. Dombroskie (2011) released a matrix key to families, subfamilies, and tribes of Lepidoptera of Canada, and Layberry et al. (1998) treated the butterflies of Canada in detail. The websites BugGuide (2018), Moth Photographers Group (2018), and Pacific Northwest Moths (Crabo et al. 2013) provide a wealth of records and information pertinent to Canada. The “Macromoths of Canada” website (Troubridge and Lafontaine 2003) has excellent images but is a little dated as it follows a previous classification scheme. Since Munroe (1979), checklists have been published for Yukon Territory (Lafontaine and Wood 1997), British Columbia (Pohl et al. 2015), Alberta (Pohl et al. 2010), Saskatchewan macromoths (Hooper 1987-2007), Ontario butterflies and macromoths (Riotte 1992), Quebec butterflies and macromoths (Handfield 1999; revised in 2011), Quebec and Labrador combined (Handfield et al. 1997), Newfoundland and Labrador butterflies and macromoths (Morris 1980), and New Brunswick butterflies and macromoths (Webster et al. 2006).

Pohl et al. (2018) published a comprehensive checklist of the Lepidoptera of Canada and Alaska, which captures information from all the above sources and others. It lists 5405 species known to occur in Canada, and an additional 50 unconfirmed records, for a total reported fauna of 5455 species in 81 families (Table 1). In total, 207 of these species (3.8%) are non-native, and 262 (4.8%) are Holarctic. Pohl et al. (2018) provides a detailed review of historical literature, as well as an extensive list of taxonomic works relevant to the Canadian fauna and a list of collections with Canadian holdings. Thus, our knowledge of the distribution of Lepidoptera in Canada has been comprehensively detailed there, and the present paper essentially compares Munroe (1979) to Pohl et al. (2018).

**Table 1.** Census of Lepidoptera in Canada. Source for currently known and introduced species is Pohl et al. (2018).

Taxon^1^	No. species reported in Munroe (1979)^2^	No. species currently known in Canada^3^	No. BINs available for Canadian species	Est. no. undescribed or unrecorded species in Canada	General distribution by ecozone^3A^
**Superfamily Micropterigoidea**					
Micropterigidae	2	2	4	0	south of taiga ecozones
**Superfamily Eriocranioidea**					
Eriocraniidae	2	2	6	2	south of taiga ecozones
**Superfamily Hepialoidea**					
Hepialidae	10	13 (1)	11	1	south of taiga ecozones
**Superfamily Neopseustoidea**					
Acanthopteroctetidae	0**^4^**	2	2	1	south of taiga ecozones
**Superfamily Nepticuloidea**					
Nepticulidae	38	69 (9)	110	50	all except Arctic
Opostegidae	4	4	14	10	south of taiga ecozones
**Superfamily Adeloidea**					
Prodoxidae	1 (16)**^5^**	22 (1)	23	2	all ecozones
Tridentaformidae	0**^6^**	1	0	0	Pacific Maritime, Prairies
Incurvariidae	33 (2)**^7^**	2	6	4	south of taiga ecozones
Heliozelidae	15	17	23	5	south of taiga ecozones
Adelidae	0 (10)**^8^**	10	14	4	all ecozones
**Superfamily Tischerioidea**					
Tischeriidae	8	14	18	5	south of taiga ecozones
**Superfamily Tineoidea**					
Meessiidae	0	1	1	1	Mixedwood Plains
Psychidae	6	11 (4)	13	3	south of taiga ecozones
Dryadaulidae	0 (1)**^9^**	1	5	4	Mixedwood Plains, Atlantic Maritime
Tineidae	23 (22)**^10^**	62 (9)	106	50	all except Arctic
**Superfamily Gracillarioidea**					
Bucculatricidae	0 (30)**^11^**	39	69	30	all except Arctic
Gracillariidae	115**^12^**	165 (5)	237	90	all except Arctic
**Superfamily Yponomeutoidea**					
Yponomeutidae	21 (19)**^13^**	19 (7)	22	5	all except Arctic
Ypsolophidae	0 (12)**^14^**	15 (2)	20	5	south of taiga ecozones
Plutellidae	18 (6)**^15^**	11	13	5	all ecozones
Glyphipterigidae	3**^16^**	15 (1)	13	5	south of taiga ecozones
Argyresthiidae	23	33 (2)	42	10	all ecozones
Lyonetiidae	40 (9)**^17^**	11 (2)	14	5	south of taiga ecozones
Attevidae	0 (1)**^18^**	1	1	0	Mixedwood Plains, Atlantic Maritime
Praydidae	0 (1)**^19^**	2 (1)	2	0	Montaine Cordillera, Mixedwood Plains, Newfoundland Boreal
Heliodinidae	5 (3)**^20^**	4	3	1	Montaine Cordillera, Prairies, Mixedwood Plains
Bedelliidae	0 (1)**^21^**	1	1	0	south of taiga ecozones
**unassigned superfamily**					
unassigned Apoditrysia	0**^22^**	1	0	0	Mixedwood Plains
**Superfamily Douglasioidea**					
Douglasiidae	4	5	7	3	all except Arctic
**Superfamily Gelechioidea**					
Autostichidae	0 (5)**^23^**	7 (1)	10	5	south of taiga ecozones
Lecithoceridae	0	1	0	0	Mixedwood Plains
Oecophoridae	79 (14)**^24^**	20 (5)	25	10	all except Arctic
Depressariidae	0 (64)**^25^**	87 (9)	88	5	all except Arctic
Cosmopterigidae	10	29	48	20	south of taiga ecozones
Gelechiidae	525 (200)**^26^**	370 (14)	604	350	all ecozones
Elachistidae	28**^27^**	66 (2)	95	50	all ecozones
Coleophoridae	55 (51)**^28^**	109 (10)	210	150	all except Arctic
Batrachedridae	0 (4)**^28^**	6 (1)	8	2	south of taiga ecozones
Scythrididae	15	14 (1)	28	15	all except Arctic
Blastobasidae	20 (17)**^29^**	19	41	30	south of taiga ecozones
Stathmopodidae	0	1	1	1	Boreal Shield, Newfoundland Boreal, Mixedwood Plains, Atlantic Maritime
Momphidae	10	24 (3)	54	30	all except Arctic
Pterolonchidae	0	2 (1)	1	0	Pacific Maritime, Boreal Cordillera
Lypusidae	0 (1)**^30^**	1 (1)	1	0	Pacific Maritime
**Superfamily Alucitoidea**					
Alucitidae	1	3	4	0	all except Arctic
**Superfamily Pterophoroidea**					
Pterophoridae	50	82 (1)	85	15	all ecozones
**Superfamily Carposinoidea**					
Copromorphidae	1	2	2	0	Montane Cordillera
Carposinidae	4	4	8	1	south of taiga ecozones
**Superfamily Schreckensteinioidea**					
Schreckensteiniidae	0 (2)**^31^**	3	5	0	all except Arctic
**Superfamily Epermenioidea**					
Epermeniidae	4	8	12	2	south of taiga ecozones
**Superfamily Urodoidea**					
Urodidae	0	1	1	0	south of taiga ecozones
**Superfamily Choreutoidea**					
Choreutidae	7	19 (1)	23	10	all except Arctic
**Superfamily Galacticoidea**					
Galacticidae	0	1 (1)	0	0	Mixedwood Plains
**Superfamily Tortricoidea**					
Tortricidae	556**^32^**	835 (41)	791	100	all ecozones
**Superfamily Cossoidea**					
Cossidae	5	6 (1)	6	0	all except Arctic
Sesiidae	44	62 (4)	50	14	all except Arctic
**Superfamily Zygaenoidea**					
Limacodidae	14	18	13	0	south of taiga ecozones
Zygaenidae	1	3	2	0	Atlantic Maritime, Mixedwood Plains, Boreal Plains
**Superfamily Thyridoidea**					
Thyrididae	3	2	1	1	south of taiga ecozones
**Superfamily Papilionoidea**					
Hesperiidae	64	74 (1)	55	5	all ecozones
Papilionidae	18	18	14	0	all ecozones
Pieridae	37	42 (1)	20	1	all ecozones
Lycaenidae	58	66 (1)	55	1	all ecozones
Riodinidae	1	1	2	1	Montane Cordillera, Prairies
Nymphalidae	94**^33^**	105 (1)	93	4	all ecozones
**Superfamily Pyraloidea**					
Pyralidae	400 (175)**^34^**	243 (15)	203	30	all ecozones
Crambidae	0 (225)**^34^**	295 (7)	295	40	all ecozones
**Superfamily Mimallonoidea**					
Mimallonidae	2	2	1	0	Mixedwood Plains
**Superfamily Drepanoidea**					
Drepanidae	18 (12)**^35^**	12	12	1	south of taiga ecozones
**Superfamily Lasiocampoidea**					
Lasiocampidae	10	8	18	2	all except Arctic
**Superfamily Bombycoidea^36^**					
Apatelodidae	2	2	1	0	Mixedwood Plains
Saturniidae	23	24 (1)	12	0	south of taiga ecozones
Sphingidae	54	60 (3)	47	3	all ecozones
**Superfamily Geometroidea**					
Uraniidae	2	2	5	0	all except Arctic
Geometridae	450	534 (9)	559	50	all ecozones
**Superfamily Noctuoidea**					
Notodontidae	50	57	64	10	all except Arctic
Erebidae	86 (286)**^37^**	342 (7)	318	40	all ecozones
Euteliidae	0 (5)**^38^**	8	6	0	Montane Cordillera, Prairies, Mixedwood Plains, Atlantic Maritime
Nolidae	0 (15)**^39^**	18 (2)	19	0	all except Arctic
Noctuidae	1520 (1050)**^40^**	1182 (18)	998	100	all ecozones
[unknown Lepidoptera]			28		
**Total**	**4692 (4107)^41^**	**5455 (207)**	**5842**	**1400**	

**^1^**Classification follows Pohl et al. (2018). **^2^**Numbers in brackets are corrections for tabulation errors and taxonomic changes, detailed in the footnotes below. **^3^**Numbers in brackets are non-native species. **^3A^** See figure 1 in Langor (2019) for a map of ecozones. **^4^**Munroe treated this family as part of Eriocraniidae. No Canadian species were known at that time, although one was listed as expected. **^5^**Munroe reported only the Yucca Moth (*Tegeticulayuccasella* Riley) from Canada for this family, but we have added 15 known species transferred from the Incurvariidae. **^6^**Munroe predicted that the sole described species, *Tridentaformafuscoleuca* (Braun), would be found in Canada, but it was placed in the Incurvariidae at that time. **^7^**Munroe erred in his count of this family; only 27 described species were known in Canada at the time, in what then comprised the Incurvariidae, so we have reduced the count by six. As well, we have removed 10 species that are now placed in Adelidae, and 15 species now placed in Prodoxidae, leaving only two species remaining in Incurvariidae, that were known in 1979. **^8^**This family was treated as a subfamily of the Incurvariidae by Munroe, although the genus *Cauchas* (now in Adelidae) was placed in the Incurvariinae at that time. Munroe specifically mentions six “Adelinae” species in Canada but we have also transferred four species of *Cauchas* that were known from Canada at that time. **^9^**One species of Dryadaulidae, *Dryadaulavisaliella* (Chambers), has been known in Canada at least as long ago as Forbes (1923). It would have been counted in Tineidae by Munroe (1979). **^10^**Munroe treated the subfamily Acrolophinae (excluding *Amydria*) as a separate family; he listed no known Canadian species and ten expected “Acrolophidae” species. One species of “Tineidae” sensu Munroe has been transferred to Dryadaulidae. **^11^**Munroe treated this group as part of the Lyonetiidae. He specifically mentioned 30 known Canadian species of *Bucculatrix*, the sole genus in the family Bucculatricidae. **^12^**Munroe listed five “Phyllocnistidae”, as a separate family. They are now considered a subfamily within the Gracillariidae. **^13^**Munroe treated the Ochsenheimeriinae as a separate family, in the Tineoidea, but listed no known Canadian species. The sole species he listed as “expected”, *Ochsenheimeriavacculella* von Röslerstamm, was confirmed for Canada as the manuscript went to press, and was added as a footnote; we have included it in his species tally here. He treated the Attevidae and Praydidae as part of the Yponomeutidae; we have removed the sole known Canadian species of Attevidae, *Attevaaurea* (Fitch), which Munroe specifically mentioned in the text. We have also removed one species of Praydidae, *Praysatomocella* (Dyar), which Munroe is assumed to have been aware of, as specimens from 1927 are in the CNC where he worked. **^14^**Munroe treated this family as part of the Plutellidae; we have transferred 12 species here that we estimate would have been known from Canada at the time. **^15^**An estimated six species of Plutellidae, as currently defined (excluding 12 species now placed in Ypsolophidae), would have been known in Canada at the time. **^16^**Munroe treated Acrolepiinae as a separate family and listed one known Canadian species. **^17^**Munroe included the Bucculatricidae and Bedelliidae within this family. We have removed the 30 Canadian species of *Bucculatrix* and sole Canadian species of *Bedellia* that were specifically mentioned by Munroe in the text. **^18^**This family was not recognised at the time of Munroe. However, he specifically mentioned the sole known Canadian species, *Attevaaurea* (Fitch), which was placed in the Yponomeutidae at the time. **^19^**This family was placed within the Yponomeutidae at the time of Munroe. He did not specifically mention any species currently placed in Praydidae, but one of them, the Hop-tree Borer (*Praysatomocella* (Dyar)), is represented by Canadian specimens in the Canadian National Collection where Munroe worked, so we assume it was known to him and we have transferred it here. **^20^**Munroe included the Schreckensteiniidae within this family; we have removed two *Schreckensteinia* species that were well known in Canada at the time (reported by ESBC 1906 and Prentice 1965). **^21^**Members of this recently recognized family were placed in the Lyonetiidae at the time of Munroe. He specifically mentions the sole known Canadian species, *Bedelliasomnulentella* (Zeller), and we have transferred it here. **^22^**This unnamed group is a holding place for the genus *Cycloplasis*. It has historically been placed in the Heliodinidae, but was excluded from that group by Hsu and Powell (2004), and was further excluded from the Yponomeutoidea by Sohn et al. (2013). No Canadian species were known at the time of Munroe (1979). **^23^**Munroe placed most of the current members of this family in the subfamily Symmocinae in the Blastobasidae. Three species then placed in Symmocinae would have been known from Canada at that time: *Oegoconiadeauratella* (Herrich-Schäffer) was listed from Alberta by Bowman (1951); *Gerdanacaritella* Busck was reported from British Columbia by Clarke (1941); and *Glyphidoceraseptentrionella* Busck was described from British Columbia in 1904. Thus, we have transferred three species from Blastobasidae to Autostichidae here. Additionally, two species of the genus *Taygete* are represented in the Canadian National Collection and would have been available to Munroe; they have been transferred here from the Gelechiidae, following a recent taxonomic move of this genus (B Landry 2002). **^24^**Historically this was a much more diverse group, but most subfamilies have been removed recently. Munroe (1979) reported 52 Depressariinae, seven Ethmiinae, and four Stenomatinae (=”Stenominae” of Munroe) species, all of which have since been transferred to the Depressariidae. The species *Carcinaquercana* (Fabricius), formerly in the Oecophorinae, was also transferred to the Depressariidae. The presence of that introduced species in Victoria, British Columbia, was well known at that time (Hodges 1974). Thus we have transferred 64 species from Oecophoridae to Depressariidae. Additionally, the Chimabachinae (one species reported by Munroe) was moved to the Lypusidae. Thus from Munroe’s count, only 14 species remain in the Oecophoridae as currently constituted. **^25^**This family was not recognized at the time of Munroe; it was erected recently for groups that were previously placed in the Oecophoridae; see footnote under that family. **^26^**Munroe’s counts of 525 known and 525 expected species of Gelechiidae is wildly inaccurate. After detailed examination of literature and collections, we estimate that approximately 200 Gelechiidae species would have been known in Canada at the time, see discussion in the text. **^27^**Munroe treated the subfamily Parametriotinae as the separate family Agonoxenidae, and listed two known Canadian species. **^28^**The Batrachedridae have recently been removed from Coleophoridae, and recognised as a distinct family. Munroe (1979) specifically mentioned four known Canadian species. **^29^**We have removed the three species of Autostichidae, which Munroe treated within the subfamily Symmocinae in the Blastobasidae. **^30^**This family was not recognised in North America at the time of Munroe. The subfamily Chimabachinae (=”Cheimbachinae” [sic] Munroe) was transferred from the Oecophoridae to this family recently. The sole North American species, *Dasystoma* (=”*Cheimophila*”) *salicella* (Hübner), was specifically mentioned as occurring in Canada by Munroe. **^31^**Munroe treated the members of this family within the Heliodinidae. We have transferred the two species that were known in Canada at that time. **^32^**Munroe treated the tribe Cochylini as a separate family, with 46 species. **^33^**Munroe treated the Libytheinae (one species), Danainae (one species), and Satyrinae (30 species) as separate families. **^34^**Munroe combined the Pyralidae and Crambidae in his count, and gave no specific numbers for Canadian species known among the various subfamilies recognized at that time. We have estimated the numbers in these families as presently constituted. **^35^**Munroe made an error here; the 12 Canadian species have been well known for many decades and have not been reclassified in more than 100 years. **^36^**Munroe listed the Bombycidae species *Bombyxmori* (Linnaeus), the Silkworm Moth, known only in captivity. We have excluded it from his tabulation and our count here. **^37^**Munroe listed 16 Lymantriidae and 70 Arctiidae; both are now subfamilies of Erebidae. All other Erebidae (as currently recognized) were placed in Noctuidae at the time. We estimate that approximately 200 Erebidae other than Lymantriinae and Arctiinae would have been known in 1979, for a total of 286 species of Erebidae. **^38^**The Euteliidae were considered a subfamily of Noctuidae at the time of Munroe. He gave no details at the subfamily level within the “Noctuidae”, but our data indicates that five species now placed in Euteliidae were known in Canada at the time. **^39^**The Nolidae were considered a subfamily of Noctuidae at the time of Munroe. He gave no details at the subfamily level within the “Noctuidae”, but our data indicates that 15 species now placed in Nolidae were known in Canada at the time. **^40^**Munroe made a significant error here, but it is obscured by changes in taxonomy. We estimate that in 1979, approximately 1270 “Noctuidae” (sensu Munroe) were known from Canada, 250 fewer than he reported; see discussion in the text. Additionally, following the current classification, we have transferred an estimated 200 “Noctuidae” species to the Erebidae, five species to Euteliidae, and 15 species to Nolidae, leaving an estimated 1050 Noctuidae (as currently constituted) known in Canada at the time. **^41^**This corrected total number of Lepidoptera species known in Canada in 1979 takes into account Munroe’s errors noted above: -6 Incurvariidae, -323 Gelechiidae, -6 Drepanidae, and -250 “Noctuidae”, for a total that is 585 fewer than Munroe reported. As well, we have included the Yponomeutidae species *Ochsenheimeriavacculella*, which Munroe added in a footnote, and we have excluded the Bombycidae species *Bombyxmori*, known only in captivity.

At the time of Munroe (1979), the most recent North American checklist would have been McDunnough (1938, 1939), but it is clear from Munroe’s text that he had a working copy of the forthcoming North American list, of which he was a co-author (Hodges et al. 1983; the checklist was essentially complete in 1978 and took five years to publish). Thus, the classification and placement of most species in Munroe’s scheme can be deduced from Hodges et al. (1983). Furthermore, using the data compiled by Pohl et al. (2018), which includes hundreds of literature sources as well as collection data, we have determined what would have been known about current taxonomic groupings in Munroe’s time. We have used that information to reassess Munroe’s family-level counts in terms of the classification in use today (Table 1). His species counts for most of the families are reasonably accurate, given the classification in use at the time. However, his numbers for the Gelechiidae and Noctuidae are far off the mark, and it is clear from Munroe’s text that they were only estimates, see further discussion of those groups below. Additional small errors were made in Incurvariidae and Drepanidae. Consequently, once errors are taken into account, we estimate that rather than Munroe’s report of 4692 species, only 4107 Lepidoptera species were known in Canada in 1979.

For the DNA barcode data, all available Canadian Lepidoptera records were extracted from the Barcode of Life Datasystems (BOLD) database (Ratnasingham and Hebert 2007) on 30 September 2017. A total of 148,314 barcoded Canadian specimens belonging to 5842 distinct clusters, hereafter referred to as Barcode Index Numbers (BINs) (Ratnasingham and Hebert 2013), were found. Of those records, 145 specimens were not identified beyond “Lepidoptera”, but most of those matched BINs that had some level of identification based on other specimens in the dataset. Only 38 specimens, belonging to 28 BINs, could not be identified to family level and were excluded from further analysis. The resulting dataset thus contained 148,276 specimens, belonging to 5814 BINs, all identified at least to family level (Table 1). The numbers of undiscovered species for each family presented in Table 1 were estimated based on barcode data, augmented by the authors’ collective knowledge of existing undescribed species, the extent of sampling, and the rate of discovery of new species in the various families.

## Overview of Canadian Lepidoptera superfamilies

### Superfamily Micropterigoidea

The sole family, Micropterigidae, includes primitive moths with functional mandibles. They were revised recently for North America, and one new species was described from western Canada (Davis and Landry 2012). The new species was known at the time of Munroe (1979), but had been misidentified, so the number of known Canadian species has not changed since then (Table 1). The DNA barcode data indicate three BINs with >2% difference within *Epimartyriaauricrinella* Walsingham, but this was examined in detail by Davis and Landry (2012) and they found no morphological evidence for cryptic species, and concluded this was simply high intraspecific variation. These moths are inconspicuous and are not attracted to lights, so they are poorly collected, and their distribution in Canada is incompletely known.

### Superfamily Eriocranioidea

The sole family is the Eriocraniidae, and since Munroe (1979) the Acanthopteroctetidae have been removed from it (see below). At least two more eriocraniid species are expected in Canada, which may account for some of the additional BINs (Table 1).

### Superfamily Hepialoidea

The sole family Hepialidae (ghost moths) are medium-sized to large moths whose larvae bore into stems or roots. Currently 13 species are known from Canada, compared to 10 reported by Munroe (1979), and it is expected that one additional species will eventually be found (Table 1). The group remains in need of modern taxonomic treatment. A global catalogue with a bibliography was published by Nielsen et al. (2000).

### Superfamily Neopseustoidea

The sole Nearctic family Acanthopteroctetidae was recently recognised as distinct from the Eriocraniidae. At the time of Munroe (1979), no species were known in Canada, but two species have since been collected, one of which was described as new by Davis (1984). One additional species of *Acanthopteroctetes*, known from Glacier National Park, Montana, is expected in Canada (Table 1). Two BINs are reported for Canada (Table 1). These moths are not attracted to light and are rarely collected so Canadian distribution is incompletely known.

### Superfamily Nepticuloidea

The Nepticulidae and Opostegidae belong to this superfamily. Our knowledge of Nepticulidae has improved substantially in recent years. Munroe reported 38 species for Canada, based on Wilkinson and Scoble (1979), which was in press at the time, but the total has now nearly doubled to 69 species (Table 1). The number of BINs is much higher than the number of known species, which signals that many more species remain to be documented in Canada – we estimate 50 (Table 1). A world catalogue was published recently by van Nieukerken et al. (2016), which presents a revised higher classification scheme for the group. Due to their minute size, these moths are rarely collected and much remains to be learned about this family in Canada.

Davis and Stonis (2007) published a monograph of the New World Opostegidae, but very little Canadian material was available for that work. The four species currently known in Canada were documented by Munroe (1979). No additional described species are known adjacent to Canada, but the high number of BINs (14) indicates significant undocumented diversity, and several more species (we estimate 10) are expected to be discovered here (Table 1).

### Superfamily Adeloidea

This superfamily has undergone significant reorganisation since Munroe (1979), with the Adelidae and Tridentaformidae raised to family level, separate from the Incurvariidae. As well, the Lamproniinae have been moved from the Incurvariidae to the Prodoxidae. The five families in Canada include 52 species and 66 BINs, and 15 additional species are expected (Table 1).

The family Prodoxidae is now well documented for Canada thanks to several recent revisions (e.g., Davis et al. 1992, Pellmyr 1999, Pellmyr et al. 2005) which have increased the Canadian species count to 22. Few additional species are expected to be found in the country. Two *Tegeticula* species (yucca moths) are known in Canada, and are of conservation concern because they are restricted to a few patches of native yucca (*Yuccaglauca* Nutt.; Asparagaceae) in southern Alberta.

Globally there is only one described species of Tridentaformidae, *Tridentaformafuscoleuca* (Braun). It was placed in the Incurvariidae at the time of Munroe (1979). It was unknown in Canada at that time, but was discovered since then, in British Columbia and Alberta. No BINs are available and no additional species are expected in Canada.

Munroe (1979) reported 33 species of Incurvariidae in Canada, but this was erroneous; only 27 species would have been known from Canada as the family was constituted at the time. Most of the known species have since been moved to Adelidae (10 species) and Prodoxidae (15 species). As currently constituted, only two Incurvariidae species are recorded from Canada, both of which were known in 1979. Four more species are expected, based on DNA barcode data.

There has been no recent research on Heliozelidae in Canada; several more BINs than described species are known, and five more species are expected here.

The Adelidae were recognised as a distinct family recently, since Munroe (1979). A few undescribed species are known to occur in Canada, but there has been no taxonomic work on the group since Powell (1969).

### Superfamily Tischerioidea

Tischeriidae is the sole family in this group. Munroe (1979) reported eight species for Canada and this has now increased to 14 through recent collecting efforts (Table 1). Puplesis and Diškus (2003) published a world catalogue, erecting one new genus that occurs in Canada. These moths are rarely collected and poorly known, and it is expected that a few additional species will eventually be found in Canada, in part based on the fact that there are more BINs than documented species in the country (Table 1).

### Superfamily Tineoidea

This superfamily has undergone reorganisation recently, with Acrolophinae relegated to subfamily status in Tineidae, and Meessiidae and Dryadaulidae newly recognised as families distinct from Tineidae. The four families in Canada total 75 species and 125 BINS, and an additional 58 species are expected (Table 1).

No species now placed in Meessiidae were known from Canada at the time of Munroe (1979), but since then one has been discovered and one more, which occurs close to the Canadian border in Maine, is expected in eastern Canada. A BIN has been assigned to the single Canadian species.

The number of Psychidae species known in Canada has nearly doubled since Munroe (1979), from six to 11. This group contains a relatively high proportion of non-native species, presumably due to parthenogenetic reproduction in some taxa which enhances their colonising abilities (Davis 1964). Two of the newly recorded taxa are non-native European species, bringing the total number of non-native species to four for Canada. It is expected that three additional species will be found in Canada. Sobczyk (2011) published a world catalogue that included many new synonyms, status changes, and new combinations. The higher classification proposed therein applied only to European taxa, so the placement of many Nearctic genera remains uncertain.

Two described species of Dryadaulidae occur in the Nearctic region, one of which, *Dryadaulavisaliella* (Chambers), is known from eastern Canada; it was counted among the Tineidae by Munroe (1979). This species exhibits significant genetic variation, based on DNA barcoding, and likely represents three taxa. As well, DNA barcode data suggests that two additional species likely occur in Canada, probably undescribed.

The Tineidae, now including Acrolophinae, are much more diverse than was known at the time of Munroe (1979), now with 62 known species and approximately 50 more expected. The family includes nine non-native species, some of which are stored product pests. Little taxonomic work has been done on the family in the past 100 years, other than the higher-level work of Regier et al. (2015a). The family is in need of modern taxonomic treatment.

### Superfamily Gracillarioidea

This superfamily was recently recognised as distinct, and includes groups previously placed in the Tineoidea. Bucculatricidae were treated by Munroe (1979) within the family Lyonetiidae, and comprised 30 of the 40 Canadian “Lyonetiidae” species reported therein. Little taxonomic work has occurred on this family in North America since Braun (1963); however, an additional nine species have been added to the Canadian fauna since 1979 (Table 1). This group remains poorly known. As indicated by DNA barcode data, at least 30 more species remain undiscovered in Canada (Table 1).

Gracillariidae is the largest family in the Gracillarioidea. Munroe (1979) treated Phyllocnistinae as a separate family with five species. As constituted today, 165 species of Gracillariidae are known with an estimated 90 more species yet to be discovered (Table 1). Currently, 237 BINs are available for this family in Canada (Table 1), which supports the high estimate of undocumented species. Although a few gracillariid groups were revised recently, and some life history information has been published (e.g., Eiseman 2018), the family remains relatively poorly known. De Prins and De Prins (2005) published a world catalogue, which has been updated and made available online (De Prins and De Prins 2016). A higher-level phylogenetic analysis was published by Kawahara et al. (2017).

### Superfamily Yponomeutoidea

This superfamily was redefined recently by Sohn et al. (2013), based on molecular analyses, and currently contains 10 named families in Canada. Based on that analysis, Ochsenheimeriinae has been relegated to subfamily status within Yponomeutidae, and Attevidae and Praydidae – formerly recognised as subfamilies of Yponomeutidae – are now recognised as distinct families. As well, Ypsolophidae was separated from the Plutellidae, Acrolepiinae was added to Glyphipterigidae, and Lyonetiidae (after removal of several other groups) was moved here from Tineoidea. As well, Bedelliidae was recognised as distinct from the Lyonetiidae. Despite these major changes, we have been able to reconcile Munroe’s (1979) species tallies with the current classification (Table 1), thanks to his detailed synopses of each subfamily in the text. The yponomeutoid families Yponomeutidae, Ypsolophidae, Argyresthiidae, Lyonetiidae, and Bedelliidae have not been examined at the species level for many decades, and are in need of modern treatment. The lack of identification tools has impeded our accrual of knowledge about them, although a few species have been added to these families since 1979 (Table 1).

Our understanding of Glyphipterigidae has improved tremendously since 1979; Heppner (1985) revised the North American Glyphipteriginae, and Landry (2007) revised the genus *Acrolepiopsis*, which includes all Canadian members of the subfamily Acrolepiinae. As a consequence of that recent taxonomic research, the number of species (three) reported by Munroe (1979) has increased five-fold to 15, and it is expected that five more species will eventually be documented (Table 1). A world catalogue of the Acrolepiinae was published by Gaedike (1997).

The number of recorded species of Plutellidae, as currently defined, has almost doubled in Canada since 1979, increasing from six to 11 species (Table 1). A few more species remain to be discovered, including apparently undescribed species collected in arctic and alpine habitats.

The sole Canadian Attevidae species, *Attevaaurea* (Fitch), is native to southern Texas but it has adapted to feed on the non-native Tree-of-Heaven (*Ailanthusaltissima* (Mill.) Swingle; Simaroubaceae) that has become naturalised in temperate North America, and the moth has now spread as far north as eastern Canada where adults have been regularly trapped in recent years. A BIN is available for this species, and no additional species are expected in Canada (Table 1).

The sole Canadian Praydidae species is the Hop-tree Borer, *Praysatomocella* (Dyar), which was known in Canada in 1979 and would have been tabulated by Munroe among the Yponomeutidae. That species has since been listed as “Endangered” in Canada (COSEWIC 2015). A second, non-native species was reported in Canada (in British Columbia and Newfoundland) by deWaard et al. (2009). Each species has been assigned one BIN, and no additional species of this family are expected to currently occur in Canada (Table 1).

The Heliodinidae are well known, thanks to the revision by Hsu and Powell (2004) for North America. The Schreckensteiniidae have been removed from the group, to their own superfamily, since Munroe (1979).

### Unassigned Apoditrysia

The genus *Cycloplasis* is currently not assigned to any superfamily or family. It was provisionally placed immediately following the Yponomeutoidea by Pohl et al. (2016, 2018). The genus contains five species worldwide, two of which occur in North America. One of these, *C.panicifoliella* Clemens, was unknown to Munroe (1979), but it has been discovered recently in southern Ontario (Pohl et al. 2018). No other species are expected in Canada.

### Superfamily Douglasioidea

The sole family Douglasiidae was removed from the Yponomeutoidea and assigned to its own superfamily since Munroe (1979). The group was revised by Gaedike (1990), in German, but that paper does not adequately delimit the species and they remain difficult to identify. Gaedike (1990) did not add any new Canadian records, but since then a species previously known only from Europe was found in Yukon Territory (Pohl et al. 2018).

### Superfamily Gelechioidea

This superfamily has undergone significant reorganisation based on recent morphological and molecular work (Heikkilä et al. 2014, Sohn et al. 2016) and 15 families containing 756 species are currently known from Canada, with an additional 668 species anticipated (Table 1). The taxonomic definition of every family other than Gelechiidae has changed considerably in the past 20 years, and some questions remain concerning the higher level classification of this group. Although a few groups have been revised in recent years, this superfamily is the most poorly known major lineage of Lepidoptera in North America, and undescribed species probably outnumber the described species in some families.

As currently defined (Heikkilä et al. 2014), Autostichidae comprises a diverse family of several subfamilies that had previously been placed in their own families (Glyphidocerinae, Deocloninae) or in the Elachistidae, Oecophoridae, and Blastobasidae. At the time of Munroe (1979), this family was not recognised, but five species now contained within it were known in Canada. Currently seven Canadian species are known and five more species are estimated to be undocumented in the country.

Lecithoceridae is restricted to the southern Palaearctic, Africa, and Australia, except for the Nearctic genus *Martyringa* (two species) which was tentatively included in the family by Heikkilä et al. (2014). At the time of Munroe (1979), the genus was placed in the subfamily Depressariinae, in the family Oecophoridae, but neither species was known from Canada. One species has since been found in Canada (Handfield et al. 1997). No BIN is available for this species, and no additional species are expected to occur in Canada.

Since Munroe (1979), many groups have been moved out of the Oecophoridae to other families in the Gelechioidea, including to the Lypusidae and Depressariidae. As currently defined, 14 species of oecophorids were known from Canada in 1979. There are now 20 species known in Canada and it is estimated that 10 undocumented species occur in the country.

Until recently, Depressariidae was considered a subfamily of Elachistidae, but Munroe (1979) reported these species in Oecophoridae and listed 64 known Canadian species. There are currently 87 documented species in Canada. Although the family is better known than most gelechioids, some taxonomic problems remain, and five more species are expected to be discovered and described in Canada.

Munroe (1979) reported 10 species of Cosmopterigidae. Currently 29 species are known from Canada, with 20 more expected to occur, based in large part on the fact that many more BINs are known than are documented species. This group is relatively well known taxonomically but is poorly collected in Canada.

Gelechiidae is a large family of small, cryptically coloured moths. Significant taxonomic works have been published recently on the Dichomeridinae (Hodges 1986) and on the genus *Chionodes* (Hodges 1999), and a few smaller groups have been treated as well. Overall, however, the family is still poorly known with many genera poorly delimited and many species that cannot be identified without examination of type material. Hundreds of species likely await discovery in Canada. A checklist of North American species was published by Lee et al. (2009).

Munroe (1979) made a significant error in his report of 525 known plus 525 undiscovered Canadian Gelechiidae species. Based on examinations of hundreds of publications and almost all the significant Lepidoptera collections in Canada, Pohl et al. (2018) could only locate records of 370 gelechiid species. Only 287 of those had been described by 1979, and many of those have been identified in collections only within the past 20 years. We estimate that only about 200 species of gelechiids would have been known in Canada in 1979. It appears that Munroe’s value of 525 species was only a very rough guess, rather than a formal tabulation. In the text section (p. 453) he writes: “*Even an estimate of species can be based on only the roughest of guesses... much of the group is awaiting arrangement or is on loan to specialists. There are certainly several hundred species in Canada...”.* His value of 525 species was probably meant to be a rough estimate of the total (known plus expected) fauna, rather than separate counts of 525 known and 525 expected species. Note that Munroe based his Gelechioidea classification on Hodges (1978), which placed all the currently recognised families other than Gelechiidae as subfamilies or tribes within the lesser families of Gelechioidea, not within Gelechiidae (the only exception being two species of Autostichidae (genus *Taygete*) which were placed in Gelechiidae in 1979). Thus, the taxonomic composition of the family Gelechiidae has changed very little since then, and Munroe’s error cannot be attributed to subsequent taxonomic changes. Currently, 370 Gelechiidae species are known from Canada. The high number of BINs (604) indicates that there are many undescribed and unreported species, and we estimate that 350 species remain undocumented, underscoring that much work awaits before this family is well known in Canada.

The concept of the family Elachistidae has changed considerably since Munroe (1979). He treated the subfamily Parametriotinae as a separate family, under the name Agonoxenidae. Our knowledge of the Elachistinae has improved greatly in the past two decades, thanks to revisions of most groups by Kaila (1995, 1996, 1997, 1999a, b). The subfamily Parametriotinae is less well known with new species awaiting description. There have been significant advances in knowledge of the fauna, and the number of species documented in Canada increased from 28 in 1979 to 66 currently (Table 1). Nonetheless, this family is poorly collected in Canada and many new species and records remain to be discovered: we estimate 50. The fact that the number of BINs greatly exceeds the number of documented species further supports our estimate of high undocumented diversity in Canada.

A few works have been published recently on Canadian Coleophoridae, including Landry (1998b) and Landry and Wright (1993). The known species diversity in Canada has more than doubled, from 51 to 109 species since 1979; however, the group remains poorly known, and many more species await description and discovery (we estimate 150 species). Baldizzone et al. (2006) published a world catalogue of the family.

The species currently in Batrachedridae were included in the Coleophoridae by Munroe (1979). The New World species were revised by Hodges (1966) and very little work has been done on them since. Munroe (1979) specifically mentioned four known Canadian species but two more have been recorded since then.

Scythrididae was not well known at the time of Munroe (1979), who reported 15 Canadian species. The family was revised for North America by Landry (1991). Currently, 14 species are known from Canada, a reduction of one species since 1979 due to synonymy. However, it is estimated that half of the Canadian fauna remains undocumented.

The composition of the Blastobasidae has changed considerably since Munroe (1979), most notably with removal of the Autostichidae as a separate family. The last comprehensive work on Blastobasidae was by Dietz (1910), so it is in need of modern treatment. Adamski and Hodges (1996) published a nomenclature review and a checklist of the known North American species. This group is reasonably well collected, but most Canadian specimens sit unidentified in collections due to the difficulties identifying them. Currently, 19 species are known from Canada (up from 17 in 1979), but the number of BINs is twice as high as the number of known species, so many – we estimate 30 species – remain to be documented.

Until recently, Stathmopodidae was placed within the Oecophoridae, but the group was elevated to family status by Heikkilä et al. (2014). No Canadian species were known at the time of Munroe (1979). This small group is relatively well known in North America, although the sole Canadian species, *Stathmopodaaenea* (Braun), was overlooked in the North American checklist of Hodges et al. (1983). It was first reported from Canada by Handfield et al. (1997). A second species is expected in Canada as well.

Momphidae was recognised as a distinct family by Munroe (1979) and he reported ten species. This has now increased to 24. The family is poorly known and in need of revision. There are more than twice as many BINs as documented species in Canada, indicating that many more species – we estimate 30 – remain to be documented.

Pterolonchidae is a small family that was treated as a subfamily within the Coleophoridae at the time of Munroe (1979), but it had no known representatives in Canada. Since then, *Coelopoetamaiadella* Kaila was described and *Pteroloncheinspersa* Staudinger was introduced for biocontrol of knapweed (*Centaurea* spp.; Asteraceae). Only one BIN for Canadian species is available and no undocumented species are expected to occur in Canada.

Lypusidae was not recognised as a family in 1979 and the one species recorded from Canada at that time, *Dasystomasalicella* (Hübner), was placed in Oecophoridae (Munroe 1979). This non-native species is still the only representative of the family in Canada and no undocumented species are expected.

### Superfamily Alucitoidea

At the time of Munroe (1979), all Nearctic Alucitidae were thought to be a single species, *Alucitahexadactyla* Linnaeus, but Landry and Landry (2004) revised the Nearctic species and recognised three valid species, none of which is the Palaearctic *A.hexadactyla*. All three Nearctic species occur in Canada. Barcode data indicates a fourth BIN, based on a single specimen from western Canada that requires further research. Gielis (2003) published a world catalogue of Alucitoidea.

### Superfamily Pterophoroidea

There has been no comprehensive taxonomic work on the sole family Pterophoridae since Barnes and Lindsey (1921), so it is in need of modern treatment. It is particularly diverse in Cordilleran areas. Munroe (1979) reported 50 species in Canada and this has now increased to 82, with an estimated 15 species remaining to be documented (Table 1). Gielis (2003) published a world catalogue of Pterophoroidea.

### Superfamily Carposinoidea

Copromorphidae is a small family that is weakly defined; its present make-up may not stand up to future taxonomic study. The Nearctic genera have all been revised recently by Heppner (1984, 1986) and Sohn (2016), so the species are reasonably well known. Since 1979, documented Canadian diversity increased from one to two species, and no undocumented species are expected to occur in the country (Table 1).

Our knowledge of Canadian Carposinidae has not advanced significantly since Davis (1968) although Diakonoff (1989) clarified the identity of one of our species. The number of known species in Canada remains at four, the number reported by Munroe (1979). DNA barcode data indicates that one more species occurs in Canada, but the specimen has not been examined for verification. Multiple BINs occur in two species but this may refer to intraspecific variation.

### Superfamily Schreckensteinioidea

The family Schreckensteiniidae contains only eight species globally. Munroe (1979) treated the two Canadian species known at the time within the family Heliodinidae. One more species has since been discovered in Canada. The group has not been revised for many decades but all three Canadian species were treated by Forbes (1923) and are reasonably well known. However, it was only fairly recently that one of our species was recognized as Holarctic rather than non-native, based on a remote collection locality in northern Alberta (Pohl et al. 2005). Although there are more BINs available than there are documented species, it is thought that this represents intraspecific variation, and no undocumented species are expected (Table 1).

### Superfamily Epermenioidea

Epermeniidae species are rarely encountered and poorly known. The North American members of the family were revised by Gaedike (1977), in German, with an updated key by Gaedike (2008). Munroe (1979) recorded four species; currently eight species are known from Canada (Table 1). There may be cryptic species yet undiscovered in Canada, as indicated by DNA barcode data. Gaedike (1996) published a world catalogue of the family.

### Superfamily Urodoidea

The family Urodidae was unknown in Canada at the time of Munroe (1979), but Landry (1998a) presented the first Canadian records. The sole Canadian species, *Wockiaasperipunctella* (Bruand), is rarely attracted to light and consequently poorly collected, so it may be more abundant than the paucity of records suggests. No more species are expected in Canada.

### Superfamily Choreutoidea

Choreutidae is poorly known and in need of modern work. The family was treated in the Sesioidea by Munroe (1979). Manuscript names were assigned decades ago by J. Heppner to Canadian specimens in collections, but most of these are still unpublished. Rota (2011) and Rota and Wahlberg (2012) revised the higher classification of the group. Seven species were recorded by Munroe (1979) and this has since nearly tripled to 19 recorded species, with an additional 10 expected in the country (Table 1).

### Superfamily Galacticoidea

The recently recognized family Galacticidae is in need of work and its composition has not been settled. The sole Nearctic species, *Homadaulaanisocentra* Meyrick, was placed in the Plutellidae at the time of Munroe (1979). It was introduced to the USA from China in the 1940s and was reported from Canada for the first time by Pohl et al. (2018).

### Superfamily Tortricoidea

The family Tortricidae is large with about 10,900 named species worldwide (Gilligan et al. 2018). Despite the importance of many species as agricultural and forest pests, many groups within the family are not well known. The tribe Cochylini was treated by Munroe (1979) as a separate family (“Cochylidae”), but has since been recognized as a subtribe within the Tortricidae. Our knowledge of North American and Canadian Tortricidae has improved substantially in recent years, with the publication of major works on Sparganothini and Atteriini (Powell and Brown 2012), and the large eucosmine genera *Eucosma* (Wright and Gilligan 2015) and *Pelochrista* (Wright and Gilligan 2017). Other significant regional works are Miller (1987) and Gilligan et al. (2008). The Canadian fauna is now known to be significantly larger than was expected by Munroe (1979) who reported 556 species and estimated 250 undocumented species. Currently, 835 species are known and another 100 undocumented species are estimated (Table 1). Brown (2005) recently published a world catalogue, which is kept current online (Gilligan et al. 2018).

### Superfamily Cossoidea

Little taxonomic work has been done on Cossidae in North America in the past century, but the species are well known and only one has been added to the Canadian fauna since Munroe (1979). His estimate of a total expected fauna of 10 species is now thought to be too high. The current number of six species in Canada matches the number of BINs, and no other undocumented species are expected (Table 1).

Members of Sesiidae were poorly collected and not very well known at the time of Munroe (1979). The family is now much better known in Canada due to a recent revision (Eichlin and Duckworth 1988), and the identification and synthesis of sex pheromones, which have been used as attractants for collecting many species. Munroe (1979) reported 44 species and the current known fauna is 62 species (Table 1). DNA barcode data indicate that several species are likely species complexes, and will require taxonomic work before the Canadian fauna is fully understood. Thus it is expected that the Canadian fauna contains about 14 undocumented species. A world checklist was published by Pühringer and Kallies (2004) and is maintained online by Pühringer and Kallies (2015).

### Superfamily Zygaenoidea

This superfamily is highly diverse in the tropics, but only a few species in two families reach into the temperate regions of Canada. No additional species of this superfamily are expected to be found in Canada. Limacodidae has not been revised in many years but the species in North America are reasonably well known. Munroe (1979) reported 14 species and four more have been subsequently recorded in Canada (Table 1). The Nearctic genera of Zygaenidae were revised by Tarmann (1984; in German) but the species have not been revised in many years. The number of species known from Canada has increased from one in 1979 to three currently (Table 1). Munroe (1979) listed one species of Megalopygidae as expected in Canada: *Megalopygecrispata* (Packard). It occurs only as far north as southern Ohio and southern New York state, and we do not expect it to be found here.

### Superfamily Thyridoidea

Little research has been done on Thyrididae in the past century. Munroe (1979) reported three species from Canada. Two of these, *Thyrismaculata* Harris and *Pseudothyrissepulchralis* (Boisduval), are fairly well known. However, the third species, *Dysodiaoculatana* Clemens, was reported by Munroe (1979) as “a southeastern species that enters southern Ontario”, but no specimens or observation records could be located and there are no other reports of it in the Canadian literature so the record remains unverified. The mention of that species by Munroe was overlooked by Pohl et al. (2018). Thus, currently we record two species from Canada with the possibility that an additional species may eventually be found.

### Superfamily Papilionoidea

The butterflies have been treated extensively in scientific and popular literature, including a comprehensive treatment of Canadian species by Layberry et al. (1998). Butterflies are relatively well understood taxonomically, but some uncertainties exist at the species level and new species and subspecies continue to be described. There are six families in Canada for which 306 species are recorded and an additional 12 species are expected (Table 1). The relationships among butterfly families were not well understood until relatively recently, and all families are now placed in a single superfamily (Heikkilä et al. 2012). Pelham (2008) published a comprehensive catalogue, which is kept up to date online (Pelham 2016).

Hesperiidae is the most poorly known family of butterflies in Canada, as they are less often sampled or studied owing to their small size and often challenging identification. Ten species have been added since Munroe (1979), raising the national total to 74 species. Five more species are expected to be described or discovered in Canada.

Taxonomic changes in the Papilionidae have resulted in a few changed species concepts, but there are few Canadian species and the group is well-studied so there has been no change in number of species (18) since 1979. Five new species of Pieridae and eight of Lycaenidae have been recognized in the Canadian fauna since Munroe (1979), raising the totals to 42 and 66, respectively. These two families include some of the taxonomically most difficult groups, and DNA barcode diagnostic performance is especially poor for sulphurs (genus *Colias*) and blues (Lycaenidae: Polyommatinae). It is expected that one additional species of each family may occur in Canada.

There is currently a single species of Riodinidae in Canada, *Apodemiamormo* (Felder and Felder), but molecular data indicate that the British Columbia and Saskatchewan populations may in fact represent two species (Proshek et al. 2015), and this accounts for the additional expected species. Both populations are of conservation concern. Munroe (1979) listed three more species expected in Canada, possibly based on the occurrence of *Calephelis* species in northeastern USA, but the probability that any of these occur in Canada is low.

Nymphalidae is the largest butterfly family in Canada (and worldwide). Several subfamilies (Libytheinae, Danainae, and Satyrinae) were historically treated as separate families, and were listed as such by Munroe (1979). Munroe reported 94 species combined; since then, 11 more species have been discovered or recognized in Canada. One additional described species is expected to be found here, and taxonomic work may lead to a few more species being recognized here as well.

### Superfamily Pyraloidea

Munroe (1979) treated the Crambidae and Pyralidae together as the Pyralidae, and did not give details on the numbers of each subfamily (which is surprising since this was his group of expertise), so the numbers reported in 1979 for each of these families as currently constituted could only be estimated. The majority of Canadian Pyralidae species are in the subfamily Phycitinae, which are fairly well known thanks to revisions by Neunzig (1986, 1990, 1997, 2003) that cover most species in the group. However, some taxonomic issues remain, and the ranges and life histories of many species are not well known. All the other subfamilies that occur in Canada (Galleriinae, Chrysauginae, Pyralinae, and Epipaschiinae) are in need of taxonomic work. Currently, the known species richness of Canada stands at 243, and we estimate that 30 undocumented species await discovery (Table 1).

Currently, 295 species of Crambidae are recorded from Canada and we estimate that 40 more species may eventually be documented in the country (Table 1). Very little taxonomic information has been published about Canadian Crambidae since 1979, and the family remains incompletely known. The Crambinae in particular are in need of modern revision; B. Landry (1995) provided an analysis and classification of North American genera. Scholtens and Solis (2015) provided an updated checklist of North American Pyraloidea.

### Superfamily Mimallonoidea

Mimallonidae is primarily a Neotropical family, with two species that have ranges extending into southeastern Canada. No more species are expected in Canada.

### Superfamily Drepanoidea

Munroe (1979) treated Drepaninae and Thyatirinae as separate families, but these are both now in Drepanidae. He listed 14 species of Thyatirinae which is erroneous. As of 2018, there are eight known species and seven subspecies of Thyatirinae in Canada. This is a very well known group; all eight Canadian species have not changed in status for decades, and all were known in Canada in 1979. The reason for Munroe’s extra species count is not known, but a possibility is that he accidentally included subspecies names. The four Canadian species of Drepaninae have also been well known to lepidopterists for decades. Consequently, the number of species in this family remains at 12 for Canada, but one more species may eventually be recorded (Table 1).

### Superfamily Lasiocampoidea

Munroe (1979) listed 10 species of Lasiocampidae, two more than currently known; however, his count was based on a classification that considered *Malacosomapluvialis* (Dyar) and *M.lutescens* (Neumögen and Dyar) as separate species, rather than as subspecies of *M.californica* (Packard) as they are treated today. The taxonomic status of those taxa remains uncertain, and DNA barcode data indicates significant divisions within *M.californica*, contributing to the 18 BINs in total for the family. Future work may prove Munroe correct in his depiction of this group. We expect that two additional species will eventually be recognized or documented in Canada.

### Superfamily Bombycoidea

There are three families of Bombycoidea in the wild in Canada. A fourth, Bombycidae, is an Old World group, known in North America only by the domesticated silkworm moth *Bombyxmori* (Linnaeus), which is cultured in captivity. Munroe (1979) and Pohl et al. (2018) included it in their counts, but we have excluded it here.

Apatelodidae is primarily a Neotropical group, with two species that have ranges extending into southeastern Canada, and no additional species are expected to be found.

Saturniidae is a very well-known family in North America. Tuskes et al. (1996) added much to our knowledge of the biology of the group, but did not add any new Canadian records. Munroe (1979) reported 23 species in Canada, but Pohl et al. (2018) list one more species: an old record of *Coloradiapandora* Blake from Victoria, British Columbia, that is treated therein as a naturally-occurring stray.

Sphingidae are very well known in Canada, based on detailed treatments of the North American fauna (e.g., Tuttle 2007). Six species have been added to the known Canadian fauna in recent years, so the current number of known species is 60, but three more species may yet be discovered in the country (Table 1). Kitching and Cadiou (2000) provided a complete world catalogue.

### Superfamily Geometroidea

Of the two families of Geometroidea in Canada, Uraniidae is primarily a tropical group with only two species in Canada. No additional species are expected; additional BINs in one species appear to represent intraspecific variation. There is no modern taxonomic revision for either genus present in Canada.

Geometridae is a huge group, containing about 23,000 species globally (van Nieukerken et al. 2011). Most Canadian species are fairly well known, but undescribed Canadian species are known in several genera. Authoritative taxonomic works are Bolte (1990) and Ferguson (1985, 2008); diagnostic references by McGuffin (1967, 1972, 1977, 1981, 1987, 1988) are useful as well. Many Canadian species were described or otherwise added to our known fauna in the works published since Munroe (1979), and the current total diversity is 534 species, an increase of 84 species since 1979 (Table 1). We expect 50 more species to eventually be documented from Canada. A global catalogue of the Geometridae was published by Scoble (1999), and an updated checklist derived from it is available online (Scoble and Hausmann 2007).

### Superfamily Noctuoidea

Five families of Noctuoidea are recorded from Canada. The world catalogue of “Noctuidae” by Poole (1989) includes most species of Noctuoidea, but excludes the Notodontidae, Lymantriinae and Arctiinae. Lafontaine and Schmidt (2010, 2011, 2013, 2015) published a more recent checklist (plus errata and additions) of valid North American Noctuoidea species. Noctuoidea are particularly well-sampled for DNA barcodes in North America; Zahiri et al. (2017) report that barcodes are known for over 97% of known North American species, with far more species sharing barcodes (255) than species not sampled for barcodes (99).

Notodontidae is the most basal North American noctuoid family. Most Canadian notodontid species are fairly well-known, but the group has not been revised in many decades. A few species have been added by collectors since Munroe (1979), so the number of species has increased to 57 from the 50 reported in 1979 (Table 1). A world catalogue was published by Schintlmeister (2013).

The remainder of the Noctuoidea have undergone considerable reorganization in the past few decades. Munroe recognized “Lymantriidae” and “Arctiidae”, and placed all remaining species in the “Noctuidae”. More recently, a series of papers examining Noctuoidea phylogeny using genetic and morphological data (Zahiri et al. 2011, 2013, and references therein) has resulted in a stable classification where the family Erebidae includes the Lymantriinae and Arctiinae as well as all the “noctuid” species that exhibit primitive “quadrifine” hindwing venation. Two smaller families Euteliidae and Nolidae are also now recognized, and the rest of the more derived groups of noctuoids are placed in a more restricted concept of the family Noctuidae.

Taking into account the aforementioned classification changes, Munroe’s (1979) report of 1520 known Canadian “Noctuidae” is still incorrect. Currently, based on Pohl et al. (2018), there are only 1439 known Canadian species that would have been placed in “Noctuidae” sensu Munroe (1979). Of those 1439 currently recognized species, only 1335 had been described by 1979, and many of those had not yet been discovered in Canada. We estimate that in 1979, approximately 1270 “Noctuidae” (sensu Munroe 1979) were known from Canada. Thus Munroe’s value of 1520 is too high by approximately 250. Munroe states in the text section (pp. 279): “*There are perhaps 1500 species of Noctuidae known from Canada...*”; suggesting that his value was only a rough guess rather than based on a tabulation of species. Another possibility is that he may have inadvertently included subspecies in his counts, as 250 is approximately equal to the known Canadian subspecies of “Noctuidae” (sensu Munroe 1979) recognized at that time. Of the estimated 1270 species of “Noctuidae” (sensu Munroe 1979) that were known in 1979, five were eutelliids, 15 were nolids, approximately 200 were erebids other than Lymantriinae and Arctiinae, and approximately 1050 were Noctuidae in the modern sense.

Erebidae is the most speciose Lepidoptera family in the world, with almost 25,000 described species (van Nieukerken et al. 2011). There are no recent comprehensive revisions, but many species are covered and illustrated in field guides and other popular works. Currently, 342 species are recorded from Canada, an increase from approximately 286 species known in 1979 (Table 1). Forty more species of Erebidae are expected in Canada. A catalogue of North American Arctiinae was published by Schmidt and Opler (2008).

The Euteliidae and Nolidae are small families that are relatively well known in Canada. At the time of Munroe (1979), five species of euteliids and 15 of nolids were known from Canada. The current numbers of species are now eight and 18, respectively (Table 1). No additional species are expected for either family.

Noctuidae is the most speciose Lepidoptera family in Canada (Table 1), and the number of species has grown from approximately 1050 to 1182 species since 1979. Many works have been published on Noctuidae in recent years, increasing our understanding of the group significantly. Numerous Noctuoidea generic revisions are available in the ongoing series “Contributions to the Systematics of New World Macro-moths“ (Schmidt and Lafontaine 2015). Comprehensive works include Lafontaine and Poole (1991; Plusiinae), Poole (1995; Cuculliinae), Hardwick (1996; Heliothinae), and Lafontaine (1987, 1998, 2004; Noctuinae). A further 100 species are expected in Canada.

## Faunal analysis

### DNA barcode information

The use of mitochondrial cytochrome *c* oxidase subunit I as a “DNA barcode” for diagnostic and taxonomic work was developed in 2003 (Hebert et al. 2003). Since then, a vast library of DNA barcodes has been assembled (Ratnasingham and Hebert 2007, 2013), including many Lepidoptera species. In previous studies, deWaard et al. (2011)found 93.2% congruence between BINs and named species in a study of 339 of the 349 geometrid species then known from British Columbia. Zahiri et al. (2017) found 92.8% congruence between BINs and species of Noctuoidea in North America, covering 3565 of 3664 species. They found a total of 3816 BINs, representing a BINs: species ratio of 1.07. In both studies, some of the incongruence was attributed to groups in need of modern taxonomic revision and which contained cryptic species. However, both studies revealed species that exhibited intraspecific variation of the barcode region, and/or shared barcodes with other species, indicating that there are cases where barcodes are not diagnostic. Nonetheless, they have greater than 90% efficacy in North American Lepidoptera.

To date, 5842 BINs have been identified among Canadian Lepidoptera. Many of the barcoded specimens were not identified to species, so a measure of efficacy cannot be calculated. However, if the ratio of BINs to species is similar to that found by Zahiri et al. (2017), this would correspond to 5461 species. Although this is extremely close to the 5455 reported species in Canada, that is merely coincidence, since some named species have not been barcoded and some barcoded species have not been named. The estimate of 5461 barcoded species represents approximately 80% of the estimated 6855 species thought to occur in Canada. Thus, we extrapolate that approximately 80% of Canadian Lepidoptera have been barcoded, although not all have named species-level determinations yet.

In several superfamilies, the number of BINs is significantly greater than the known species (Nepticuloidea, Tineoidea, Gracillarioidea, Gelechioidea), reflecting our lack of knowledge in those groups (see discussion of undescribed species below). Most butterfly families are very well sampled genetically, and are very well known; the lower numbers of BINs than described butterfly species indicates cases where BINs are not diagnostic at the species level. This is most notable in the Pieridae, particularly in the genus *Colias* in which 15 species share five BINs in Canada (Ratnasingham and Hebert 2007).

### Current state of knowledge

The checklist by Pohl et al. (2018) lists 5455 species of Lepidoptera reported from Canada, making it the fourth-largest insect order in the country in terms of diversity. A total of 207 species is known to be non-native to North America, 63 of which arrived or were detected after 1979. A further 65 species (not detailed here) are of unknown origin, either non-native or Holarctic.

Our knowledge of Lepidoptera in Canada is generally good, but it is unevenly spread geographically and taxonomically. The composition and distribution of many micromoth families are relatively poorly known, while butterflies and most macromoths are relatively well known. As reported by Munroe (1979), biological and ecological information remains mostly concentrated on a few important pest species. However, the recent expansion of interest in invertebrate conservation has improved our knowledge of some species.

Munroe (1979) reported 4692 Lepidoptera species known from Canada, and estimated an additional 2042 species that likely occurred here but had yet to be discovered, for a total of 6734 species. However, after taking into account the aforementioned errors in his counts of Incurvariidae, Gelechiidae, Drepanidae, and Noctuidae, only 4107 species were known from Canada at that time, representing 67% of an estimated total fauna of 6149 species. As of 2018, 5455 species are reported from the country and a further 1400 are expected to be discovered, for an estimated total fauna of 6855 species. Since 1979, 1348 more species of Lepidoptera have been documented in Canada, representing 66% of the additional species Munroe predicted would be found here, for an increase in the known fauna of about 33%. Munroe predicted a higher number of Adeloidea than we do, and his estimates for Tineoidea, Gelechioidea, Tortricoidea, and Pyraloidea are significantly lower than ours. Currently, it is estimated that about 80% of the total Canadian species are known, a much higher proportion than in 1979. The approximately 1400 species thought to comprise Canada’s unknown Lepidoptera fauna include both species unknown to science and described species with a core range outside of Canada that have not yet been documented here. The undocumented fauna for micromoths is likely to contain a higher proportion of undescribed species than for the taxonomically better-studied macromoths. Species additions among the butterflies will likely be the result of taxonomic changes.

There have been two drivers of the increase in our knowledge of Canadian Lepidoptera. First has been the slow and steady accumulation of new knowledge – the new records, new species descriptions, and revisions, augmented with new character sets and tools such as genetic information and the analytical techniques to derive value from it. The development of DNA barcoding by the Barcode of Life Data Systems group (Ratnasingham and Hebert 2007, 2013) has helped immensely in making sense of species-level genetic variation, and it is a testament to Canadian ingenuity that such an internationally important organization was built in our country. The second driver has been a revolution in how we access existing knowledge. The modern computer and the internet age have put almost the entire written word at our fingertips, easily accessible to anyone with a computer and an internet connection. Tasks that took weeks in Munroe’s day, such as obtaining an obscure paper, or getting the opinions of colleagues across the world, now take minutes to hours. Today’s curious naturalist can post a photo of a moth online, and trigger a real-time discussion about its identity among the world’s top authorities. That was unthinkable in Munroe’s time. As well, the simple ability to organize and electronically search vast amounts of information has improved the life of the biologist immeasurably. It was simply not possible to compile a definitive checklist of the Lepidoptera of Canada before modern computers existed. Going forward, the steady accumulation of new information, and enhanced ability to access and organize vast amounts of data will continue to drive our knowledge of Canadian Lepidoptera.

## Gaps and opportunities

### Undersampled regions

Historic and modern sampling effort for Lepidoptera has not been equal across Canada’s vast landscape. Urban centres and adjacent areas with a long history of lepidopterists accordingly have the best-known Lepidoptera fauna; other regions have only a limited history of Lepidoptera surveying or are still relatively unknown. These knowledge gaps can be evaluated through the lens of either political or ecological geography at different spatial scales. Comparison of Lepidoptera diversity patterns to other well-sampled fauna and flora at the provincial/territorial scale provides a good starting point for addressing future research efforts.

As nearly all Lepidoptera depend on plants, comparison of their species richness to that of vascular plants provides a meaningful comparative metric of discrepancies in actual or observed diversity across jurisdictions. The ratio of native Lepidoptera to native vascular plant species richness is remarkably consistent across most of southern Canada, ranging from 1.42–1.60 for the Prairie Provinces, Ontario, Quebec and New Brunswick (Table 2); these regions are generally regarded as being well-known entomologically and botanically. British Columbia has a lower ratio of 1.21, and given that it has the greatest plant diversity in Canada, many more Lepidoptera likely remain to be documented there. As discussed below, British Columbia also has a high incidence of Lepidoptera species new to science. Ratios for the Maritime Provinces are more variable; New Brunswick has a well-known Lepidoptera fauna with a ratio comparable to the rest of southern Canada, while that for Nova Scotia indicates slightly greater Lepidoptera numbers (or possibly a more poorly known flora). Prince Edward Island has the lowest ratio among non-northern Canadian jurisdictions; more Lepidoptera species are expected (but not yet documented) there than in any other province (Pohl et al. 2018). A value of 0.97 for Newfoundland (excluding Labrador) is intermediate between that of southern Canada and the North, as might be predicted given its ecozonal affinities (primarily boreal with some subarctic elements), and the fact that it is well removed from the mainland and therefore largely lacking southern and Atlantic Maritime Lepidoptera. The Lepidoptera of Newfoundland has historically been relatively well studied, although even macromoth species continue to be added to the island’s fauna (B Landry and Schmidt 2018). Lepidoptera-to-plant diversity ratios are considerably lower for the North (Yukon Territory, Northwest Territories, Nunavut, Labrador) compared to southern Canada, undoubtedly reflecting different latitudinal patterns in diversity gradients between insects and plants. Nevertheless, comparisons among northern jurisdictions indicate a considerably lower value for Nunavut. The bulk of Nunavut’s Lepidoptera faunistics information is based on the Northern Insect Survey work carried out decades ago (Danks 1981), and very limited additional Lepidoptera collecting has occurred there, in large part due to the remoteness and inaccessibility of the region. The southeastern-most extent of Nunavut comprising numerous islands in Hudson Bay and James Bay (e.g., Belcher Islands, Akimiski Island) will certainly reveal species new for the territory, as virtually no sampling has occurred there. As well, the mainland portion of Nunavut that lies north of Manitoba, which contains significant areas of boreal forest, has been poorly sampled and will likely yield many new records.

**Table 2.** Comparison of number of native Canadian Lepidoptera species (Pohl et al. 2018) and native vascular plant species (Canadian Endangered Species Conservation Council 2016).

	**YT**	**NT**	**NU**	**BC**	**AB**	**SK**	**MB**	**ON**	**QC**	**LB**	**NF**	**NB**	**NS**	**PE**
No. Lepidoptera species	739	601	139	2633	2467	1880	2111	3058	2772	484	853	1593	1745	776
No. plant species	1056	1046	668	2176	1602	1230	1349	2038	1736	682	877	1125	1069	713
Lepidoptera : plant ratio	0.70	0.57	0.21	1.21	1.54	1.53	1.56	1.50	1.60	0.71	0.97	1.42	1.63	1.09

From an ecological perspective, data on the Lepidoptera fauna by ecozone is more limited. Of Canada’s 15 terrestrial ecozones (Federal, Provincial and Territorial Governments of Canada 2010), the boreal region is the most expansive (here defined to include the Boreal Shield, Newfoundland Boreal, Boreal Plains, Taiga Shield, Taiga Plains and Hudson Plains ecozones) and it has a relatively well-sampled, taxonomically well-known, homogeneous Lepidoptera fauna where many local faunal inventories have been carried out (see Pohl et al. 2010, Pohl et al. 2018). The exception is the northern reaches of this region where lack of road access has meant that much of the region is unexplored entomologically. The Atlantic Maritime ecozone is relatively well documented (Lafontaine et al. 2001), and the Prairies ecozone moderately so (Pohl et al. 2014). Most recent discoveries, including species new to science and new additions to the Canadian fauna, have come from the Mixedwood Plains (southern Ontario) and Montane Cordillera (southern British Columbia and western Alberta) ecozones, and also Taiga Cordillera and Boreal Cordillera (northern British Columbia, Yukon, and western Northwest Territories). The Pacific Maritime ecozone is most diverse and best known around its southernmost reaches (i.e., Vancouver Island and the Lower Mainland of British Columbia), while the central and northern portions have had little survey effort, and virtually none at higher elevations. Similarly, sampling density across Canada’s Arctic ecozone remains sparse. Renewed micromoth collecting efforts in southwestern Ontario near Lake Huron by one collector (K. Stead) in recent years has resulted in a surprising number of new Canadian records for species previously known only from the central USA (south of the Great Lakes and east of the Mississippi valley). Most regions of Canada are expected to yield numerous micromoth discoveries with proper sampling. Rearing and day-time collecting with sweep nets, especially in open habitats, would likely bring many discoveries.

In summary, the jurisdictions that show the greatest deficit in Lepidoptera faunal knowledge include Nunavut, Prince Edward Island, and British Columbia. The Lepidoptera fauna has not been delineated for all ecozones, but most recent discoveries stem from the southern ecozones that also include Canada’s diversity hotspots, with the important exception of the northern cordilleran ecozones that encompass parts of Beringia. Directing future sampling effort to targeted areas will provide a more complete picture of jurisdictional and ecozonal faunal inventories that will, in turn, aid decisions in managing the future of Canada’s biologically rich heritage, particularly those species of importance to humankind and those in need of conservation.

### Undescribed species

**Figure 1.**
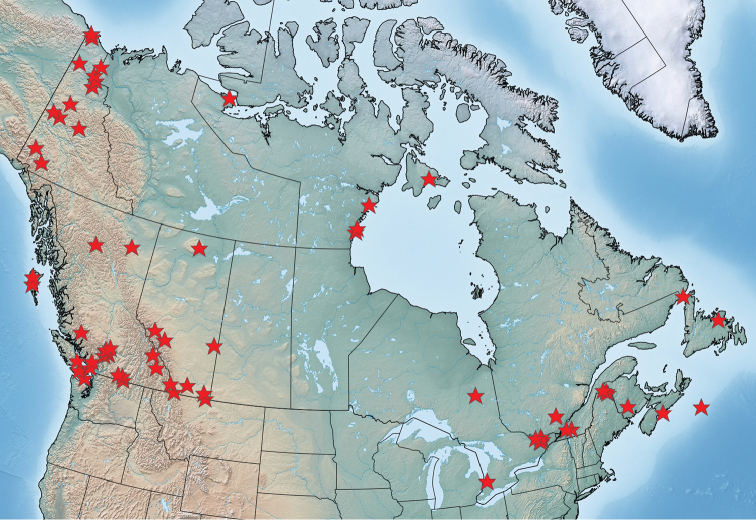
Map of all Canadian macrolepidopteran type localities for species described since 1978.

As in other insect groups, the state of taxonomic knowledge of Canada’s Lepidoptera fauna varies according to group. Butterflies are the best-known insect group taxonomically, and the few recent discoveries involve previously overlooked cryptic species (e.g., Verhulst 2009, Warren et al. 2016). Most faunal additions result from better resolution of species-groups that have traditionally been difficult to delineate, such as *Coenonymphanipisiquit* McDunnough (Sei and Porter 2007). In contrast, a moderate number of new species of macromoths continue to be discovered. Two regions stand out as recently yielding relatively high numbers of new macromoth species: unglaciated parts of the Yukon (Beringia), and the Pacific Maritime, Montane Cordillera, and Western Interior Basin of British Columbia (Fig. 1). Although few of the recently discovered species are endemic to Canada, most often occurring in neighbouring parts of the United Sates, these discoveries do highlight the importance of continued sampling and surveying of Lepidoptera, particularly in diversity hotspots such as southern British Columbia. Approximately 50 species of Canadian macromoths are currently known to be unnamed or unrecognized, primarily in the superfamily Noctuoidea but also including Geometridae (C Schmidt unpubl. data).

The micromoths are generally much less well known. As a rule, the smaller the moths, the fewer the records and the poorer the taxonomic knowledge of families. Small to minute size coupled with fragility and difficulties in specimen preparation translates into fewer, lesser-quality specimens available for study, which hinders taxonomy. Many of the smaller moths are easily missed, particularly if sampled with the usual method of light traps. Many micromoths have diurnal or crepuscular habits (e.g., Micropterigidae, Acanthopteroctetidae, Scythrididae, Epermeniidae) and are rarely collected at lights, so they are often under-represented in collections. Recent insect barcode surveys conducted across Canada with Malaise traps (Gleason and Williams 2012) yielded numerous micromoths, such as Nepticulidae, Tineidae, etc., otherwise rarely collected unless reared from larvae. In general, the proportion of undescribed species in North America exceeds 25% in many micromoth families, compared to less than 10% for the majority of macromoth groups. The superfamily Gelechioidea includes the greatest number of undescribed or unrecognized species (>650), with several families in which that portion equals or exceeds the named species (Blastobasidae 150%, Coleophoridae 138%, Gelechiidae 95%, Momphidae 125%, Scythrididae 100%; see Table 1). A similar though less extreme situation prevails in other superfamilies, notably in the Nepticuloidea, Tineoidea, and Gracillarioidea. In these taxa, the taxonomic impediment is not restricted to Canada but extends to the entire Nearctic Region.

### Distribution changes

A few species are known to have expanded their distributions since Munroe’s (1979) account. The most dramatic examples include non-native species such as *Noctuapronuba* (Linnaeus), which has spread across the continent in the past two decades after an initial introduction to Atlantic Canada (Copley and Cannings 2005, and references therein). *Lymantriadispar* (Linnaeus), the most notorious non-native lepidopteran, has not expanded into the boreal forest region, but is now widespread throughout the eastern deciduous forest. Although it is not always possible to discern whether new distribution records of native species represent range expansion or simply greater collecting effort, the detection of several conspicuous species in historically well-collected regions clearly indicate range shifts. The best-documented cases involve westward range expansions of eastern species into Alberta, such as *Actiasluna* (Linnaeus), *Acronictamansueta* (Smith), *Harrisimemnatrisignata* (Walker), *Ctenuchavirginica* (Esper), *Diachrysiaballuca* (Geyer) and *Letheanthedon* (Clarke). There are few cases of western species expanding eastward, although the western bean cutworm (*Striacostaalbicosta* (Smith)) is now regularly present in Ontario and has been found as far east as New Brunswick, but was historically restricted to the Great Plains. Northward expansions are best documented in Ontario butterflies, where *Papiliocresphontes* Cramer, *Erynnisbaptisiae* (Forbes) and *Anatrytonelogan* (Edwards) have moved north and east into the eastern part of the province in the past few decades (Larrivée and Kerr 2012). Conversely, several boreal butterfly species seem to be disappearing from some southern parts of their range. In eastern Ontario, *Coliasinterior* Scudder, *Oeneischryxus* (Doubleday), *Icariciasaepiolus* (Boisduval) and *Boloriafreija* (Thunberg) have disappeared to varying extents from historic localities. A few Canadian species have become extirpated due to habitat loss from already localized populations, such as *Plebejussamuelis* (Nabokov), *Callophrysirus* (Godart), and *Erynnispersiuspersius* (Scudder). Despite the above examples, for the most part our species-level knowledge has not been sufficient to detect and measure trends in range changes. We have not yet seen large range changes that can unequivocally be attributed to climate change, but they will undoubtedly occur. For some Lepidoptera groups such as butterflies and macromoths, we are well positioned to detect such changes; for other lesser known groups, it will be difficult to distinguish new immigrants from undetected indigenous species.

### Lepidoptera of conservation concern

A total of 26 species and an additional eight subspecies of Lepidoptera are currently ranked by COSEWIC as being of conservation concern based on detailed assessments (COSEWIC 2017). An additional 157 species have been flagged as being of potential conservation concern (ranked as “N3” or lower) at the national level by the National General Status Working Group of the Canadian Wildlife Service (CESCC 2016). A significant impediment to assigning conservation ranks to Lepidoptera is lack of knowledge about the geographic extent of occurrence and area of occupancy, both being metrics based on occurrence (or more rarely absence) records of a given species. For example, *Melaporphyriaimmortua* Grote was assessed as “data deficient” (COSEWIC 2017) since lack of knowledge about host plants and habitat requirements prevent targeted surveys for this species. Identifying common knowledge gaps among Lepidoptera of potential conservation concern would help in targeting specific regions and/or habitats for future Lepidoptera survey efforts.

### Biological knowledge

Lack of knowledge about hostplant or larval requirements can hamper our understanding of Canada’s Lepidoptera fauna, and in some cases impact management decisions, from both a conservation and pest management perspective. Lack of knowledge of basic natural history information is still a considerable data gap among Canadian Lepidoptera. Perhaps as many as half of micromoth species have completely unknown immature stages and host plant requirements. Macromoths fare somewhat better, with an estimated 30% of species that have unknown life histories. Immature stages and life histories are much better known for eastern than for western species, as the eastern fauna has been studied intensively in the past two decades (e.g., Wagner 2005, Wagner et al. 2011). The historic focus on tree and shrub insects has provided for a better understanding of the immature stages of moths that feed on such plants; for example, the Forest Insect and Disease program of the Canadian Forest Service (McGugan 1958, Prentice 1962, 1963, 1965) provided a considerable knowledge base on forest Lepidoptera biology. The least-known Lepidoptera are those that feed on rare, habitat-specialized or economically unimportant plants.

Lepidoptera is the second-most diverse group (after Hymenoptera) of flowering plant pollinators. They are closely associated with flowering plants and most Lepidopterans that imbibe nectar are potential or actual pollinators. The greatest diversity of nectarivore Lepidoptera is in the Obtectomera clade (van Nieukerken et al. 2011), notably the butterflies and skippers (superfamily Papilionoidea), owlet moths and relatives (Noctuoidea), spanworms (Geometroidea), snout moths (Pyraloidea), and hawk moths (Bombycoidea: Sphingidae). Most micromoths (non-obtectomeran Ditrysia; van Nieukerken et al. 2011) appear to play only a minor role as potential pollinators, although exceptions include the Prodoxidae, some of which are well known for their mutualisms with yucca plants.

Despite the recent focus on the importance of insect pollinators in natural and agro-ecosystems, basic data on Lepidopteran nectar-feeding ecology is so scant that it is uncertain just which Lepidoptera are pollinators, and clearly this is a research priority before the pollinator fauna can be understood. An assessment of which moth groups and which plant taxa are likely the most important players in Lepidopteran pollinator interactions in Canada is an important first step that is sorely needed.
